# Addressing health literacy gaps in adjuvant endocrine therapy adherence: Post hoc insights from a randomized remote monitoring trial

**DOI:** 10.1016/j.breast.2025.104552

**Published:** 2025-08-05

**Authors:** Ilana Graetz, Samuel Hernandez, Xin Hu, Rebecca A. Krukowski, Janeane N. Anderson, Teresa M. Waters, Edward Stepanski, Gregory A. Vidal, Lee S. Schwartzberg

**Affiliations:** a Rollins School of Public Health, Emory University, Atlanta, GA, USA; b Medical College of Georgia, Augusta, GA, USA; c School of Medicine, Emory University, Atlanta, GA, USA; d University of Virginia Comprehensive Cancer Center and School of Medicine, Charlottesville, VA, USA; e College of Nursing, University of Tennessee Health Science Center, Memphis, TN, USA; f Augusta University School of Public Health, Augusta, GA, USA; g Ovation.io, Cambridge, MA, USA; h West Cancer Center and Research Institute, Germantown, TN, USA; i Renown Institute for Cancer and University of Nevada, Reno, NV, USA

**Keywords:** Adherence, Health literacy, mHealth, Breast cancer, Endocrine therapy

## Abstract

Adjuvant endocrine therapy (AET) improves survival in hormone receptor-positive breast cancer, yet adherence is often lower among individuals with limited health literacy. This post hoc analysis of the THRIVE trial examined whether health literacy modified the effectiveness of two remote monitoring interventions (App-only and App + Feedback) versus enhanced usual care (EUC) on 12-month AET adherence (≥80 % of prescribed doses via connected pillbox). Among participants with lower health literacy, adherence was higher with App + Feedback than EUC (80.0 % vs. 42.1 %, p = 0.03), with no significant differences among those with higher health literacy. Tailored digital interventions may support adherence among patients with limited health literacy.

**Trial:**

ClinicalTrial.gov identifier NCT03592771.

## Highlights

• Lower health literacy is a barrier to endocrine therapy adherence.
• Lower health literacy was more common among Black participants.
• Monitoring app with tailored messages improved 12-month adherence among lower literacy participants.
• No significant adherence effect in higher literacy participants.
• Tailored mobile health tools have potential to reduce disparities in breast cancer outcomes.

## 1 Introduction

Despite the demonstrated efficacy of oral adjuvant endocrine therapy (AET) in improving survival rates among women with hormone receptor-positive early-stage breast cancer, adherence remains alarmingly low [1]. Many patients face challenges in maintaining the prescribed regimen for various reasons, including managing side effects, logistical barriers, and psychosocial factors [16]. Health literacy influences a patient's understanding of their treatment plans, their ability to navigate healthcare systems to access necessary support, and manage side effects, all of which are critical for treatment adherence [2]. Addressing barriers to adherence is crucial for improving patient outcomes and equity in cancer care delivery [3].

## 2 Methods

The THRIVE study (NCT03592771) evaluated whether remote monitoring interventions could improve AET adherence. In this non-blinded randomized controlled trial, consented women at a multi-site cancer center with early-stage breast cancer and recent AET prescription were randomized into (1) “Enhanced Usual Care (EUC)” reporting symptoms at each clinic visit; (2) “App-only”, receiving access to the study's adherence and symptom monitoring app for 6 months, with increasing/severe symptoms and missed doses reported in the app triggering follow-ups from the oncology team; or (3) “App + Feedback”, receiving additional weekly educational text messages about managing symptoms, adherence, and communication for 6 months.

The University of Tennessee Health Science Center Institutional Review Board approved the study, and the protocol has been previously described [4,5].

An enrollment survey captured participants’ sociodemographic characteristics (race, ethnicity, education, household income, and health literacy). Health literacy was measured using a validated single-question about how often they felt confident filling out medical forms independently [6,7]. Responses were categorized as higher (always) versus lower (never, rarely, sometimes, often) health literacy. Twelve-month AET adherence was electronically monitored using the Wisepill connected pillbox. Participants were considered adherent if they took ≥80 % of prescribed doses, excluding prescriber-advised treatment pauses and hospitalization days.

A linear probability model estimated the interaction effect between study arm and health literacy on AET adherence. Marginal effects were used to quantify differences in adherence by study arm and within each health literacy group.

## 3 Results

Among the 304 women randomized (104 EUC, 98 App-only, and 102 App + Feedback), 88 % (n = 266) completed the 12-month follow up and were included in our analyses. At baseline, 18.8 % (n = 50) reported lower health literacy (Table 1). Lower health literacy was more common among Black (29.1 %) compared to White participants (12.4 %, p < 0.001). Baseline characteristics were balanced across study arms [5].

**Table 1:** Baseline characteristics by health literacy^a^ (N = 266).

Characteristics	Lower Health Literacy (N = 50)	Higher Health Literacy (N = 216)	*P*-value
Age, years			
Mean (Standard Deviation)	58.8 (10.1)	58.9 (10.9)	0.91

**Race**, n (column %)			<0.001
Black	25 (50.0)	61 (28.2)	
White	21 (42.0)	149 (69.0)	
Other^a^	4 (8.0)	6 (2.8)	

**Education**, n (column %)			0.17
High school or less	13 (26.0)	38 (17.6)	
Some college or higher	37 (74.0)	178 (82.4)	

**Income: Federal Poverty Level (FPL)**, n (column %)			0.12
<100 % FPL	9 (18.0)	20 (9.3)	
≥100 % FPL	38 (76.0)	189 (83.3)	
Missing	3 (6.0)	7 (3.2)	

**Married or living with a partner**, n (column %)	30 (60.0)	148 (68.5)	0.25

**Location**^b^**, n (%)**			0.29
Non-metro	9 (14.3)	54 (25.0)	
Metro	41 (82.0)	162 (75.0)	

**Initial AET prescription, n (**column **%)**			0.27
Tamoxifen	15 (30.0)	49 (22.7)	
Anastrozole	33 (66.0)	145 (67.1)	
Exemestane or Letrozole	2 (4.0)	22 (10.2)	

**Cancer stage at diagnosis, n (**column **%)**			0.44
DCIS	8 (16.0)	22 (10.2)	
I	32 (64.0)	155 (71.8)	
II-III	10 (20.0)	39 (18.1)	

**Prior chemotherapy, n (**column **%)**	14 (28.6)	57 (28.1)	0.95

**Prior radiation, n (**column **%)**	29 (59.2)	136 (64.8)	0.47
**Study Arm, n (**column **%)**			0.94
EUC	19 (38.0)	78 (36.1)	
App-only	16 (32.0)	68 (31.5)	
App + Feedback	15 (30.0)	70 (32.4)	

Notes: Among participants who were randomized and completed the 12-month follow-up survey.

^2^Among participants in the ‘Other’ race category (N = 10), 5 self-identified as Asian, 1 American Indian, 1 Hispanic, and 3 as mixed race. DCIS = Ductal Carcinoma In Situ, AET = Adjuvant Endocrine Therapy, EUC = Enhanced Usual Care.

a Lower health literacy is defined as Never/Rarely/Sometimes/Often feeling confident filling out medical forms alone, while higher health literacy is defined as Always feeling confident.

b Rural-Urban Commuting Area Codes (RUCA) used to categorize residential location as metro if RUCA was 1 and non-metro if RUCA was 2–10.

**Fig. 1:**
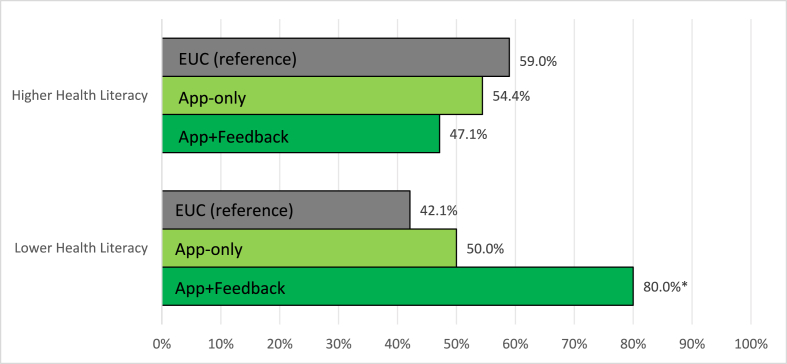
Adjusted Percentage of Participants AET Adherent by Study Arm and Health Literacy Level Note: Adjuvant Endocrine Therapy (AET) adherence was electronically monitored over 12 months by the Wisepill pillbox and calculated as the proportion of prescribed doses taken (≥80 %), excluding prescriber-advised pauses and hospitalization days. EUC = Enhanced Usual Care. ∗*P* < 0.05.

Fig. 1 displays the adjusted percentage of participants adherent to AET by study arm and health literacy level. Among those with lower health literacy, 42.1 % in the EUC arm were adherent, compared to 50.0 % in App-only (adjusted risk difference [aRD]: 7.9 percentage points; 95 % CI: 25.3 to 41.1) and 80.0 % in App + Feedback (aRD: 37.9 percentage points; 95 % CI: 4.1 to 71.7). Among participants with higher health literacy, adherence was 59.0 % in EUC, 54.4 % in App-only (aRD: −4.6 percentage points; 95 % CI: 20.8 to 11.7), and 47.1 % in App + Feedback (aRD: −11.8 percentage points; 95 % CI: 27.9 to 4.3).

## 4 Discussion

This post hoc analysis demonstrated that a remote monitoring app with tailored educational messages was associated with a substantial increase in AET adherence, from 42.1 % to 80.0 %, among women with lower health literacy, but not among those with higher health literacy. The effectiveness of the App + Feedback intervention among participants with lower health literacy underscores the value of enhanced patient-provider communication, facilitated by tailored digital tools. Simplified, accessible messaging may have enhanced patient understanding and engagement of their treatment, while app-triggered alerts facilitated timely provider communication. Together, these features may have helped bridge communication gaps that contribute to nonadherence among individuals facing multilevel barriers related to limited health literacy.

Notably, the app and messages were developed using participatory action research principles, incorporating iterative input from diverse breast cancer survivors [8]. A prior evaluation found high overall satisfaction with the remote monitoring intervention, with significantly greater satisfaction among participants with lower health literacy [9]. The disproportionate prevalence of lower health literacy among Black participants reflects structural inequities, including the effects of socioeconomic disadvantage, that adversely affect cancer care [[10], [11], [12], [13]]. Remote monitoring with tailored education offers a promising approach to mitigate health-literacy related barriers and reduce disparities in downstream cancer outcomes. Routinely assessing health literacy may help oncology practices identify patients at risk for non-adherence who could benefit from targeted remote monitoring support strategies [10].

Our finding of no impact on 12-month AET adherence [5] overall or among higher health literacy participants aligns with prior behavioral intervention trials that similarly failed to show sustained adherence improvements beyond nine months [14,15]. The relatively low adherence observed among participants with higher health literacy (47 %–59 %) is concerning but also aligns with prior studies finding AET adherence prevalence ranging from 41 % to 72 % [16]. That digital remote monitoring with educational messages improved adherence among participants with lower, but not higher, health literacy underscores the importance of identifying and addressing subpopulation-specific barriers. Thus, different tailored strategies may be needed to support adherence among patients with higher health literacy.

Despite the study's rigorous randomized design, several limitations should be considered. Since this post hoc analysis was not pre-specified and had a small sample size of participants with lower health literacy, findings should be interpreted with caution. Adherence was assessed over a 12-month period, whereas endocrine therapy is typically prescribed for five years or longer. Additionally, our study was limited to English-speaking participants with access to a smartphone, all receiving care at a cancer center that routinely monitored patient-reported symptoms during clinic visits. Consequently, the findings may not generalize to other healthcare settings or patient populations. Future studies with a larger sample of lower health literacy participants and longer follow-up period are needed to confirm these findings and determine whether early improvements in adherence are sustained longer-term.

This post hoc analysis suggests that a remote monitoring app with tailored educational messages is associated with substantially higher 12-month AET adherence among women with lower health literacy, with no effect for those with higher health literacy. Our findings underscore the importance of tailoring interventions to patient needs, particularly by enhancing communication and empowering individuals with lower health literacy, to support sustained adherence and long-term treatment success. Further research is needed to refine these approaches and evaluate their longer-term effectiveness across diverse populations.

## CRediT authorship contribution statement

**Ilana Graetz:** Writing – review & editing, Writing – original draft, Project administration, Investigation, Funding acquisition, Formal analysis, Data curation, Conceptualization. **Samuel Hernandez:** Writing – review & editing. **Xin Hu:** Writing – review & editing, Methodology, Data curation. **Rebecca A. Krukowski:** Writing – review & editing, Project administration, Conceptualization. **Janeane N. Anderson:** Writing – review & editing, Project administration, Data curation. **Teresa M. Waters:** Writing – review & editing, Project administration, Conceptualization. **Edward Stepanski:** Writing – review & editing, Project administration, Conceptualization. **Gregory A. Vidal:** Writing – review & editing, Project administration, Conceptualization. **Lee S. Schwartzberg:** Writing – review & editing, Project administration.

## Ethical approval

The University of Tennessee Health Science Center Institutional Review Board approved the study.

## Data sharing statement

The data that support the findings of this study are available from the corresponding author with IRB approval upon reasonable request.

## Funding

The National Cancer Institute funded the study (R01CA218155, PI: Graetz). The funding agency had no role in the design and conduct of the study.

## Declaration of competing interest

Dr Graetz received research grants from Pfizer and PRIME Education, LLC outside the submitted work. Dr. Hu received research support from PhRMA Foundation, St. Jude Children's Research Hospital, Johnson & Johnson, and Pfizer outside the submitted work. Dr. Anderson received research grants from NIH and Gilead Sciences, Inc. outside the submitted work. Dr. Waters received research support from NIH outside of the submitted work. Dr. Krukowski received research support from NIH, the Jeffers Trust, and PCORI outside of the submitted work. Dr. Vidal reported receiving personal fees from Roche/Genetech, Eli Lilly, Pfizer, AstraZeneca and research funding from Roche/Genetech, Puma, Celcuity, Merck, BMS, Eli Lilly, Astrazeneca, Gilead Sciences, Inc., GSK, Natera, Pfizer, Eisai and ownership of Veris Health outside the submitted work. Dr. Schwartzberg reported receiving personal fees from Pfizer, Helsinn, AstraZeneca, Genentech, Myriad, Napo, Eli Lilly, Coherus Biosciences, Foundation Medicine, Novartis, Daiichi Sankyo, Merck and GlaxoSmithKline. Sanofi, Mirati Therapeutics, GlaxoSmithKline, and research support from Amgen, Pfizer outside the submitted work. No other disclosures were reported.
